# Quantification of Mobile Ions in Perovskite Solar Cells with Thermally Activated Ion Current Measurements

**DOI:** 10.1021/acsenergylett.5c02224

**Published:** 2025-12-12

**Authors:** Moritz C. Schmidt, Agustin O. Alvarez, Riccardo Pallotta, Biruk A. Seid, Jeroen J. de Boer, Jarla Thiesbrummel, Felix Lang, Giulia Grancini, Bruno Ehrler

**Affiliations:** † LMPV - Sustainable Energy Materials Department, 55952 AMOLF, Science Park 104, 1098 XG, Amsterdam, The Netherlands; ‡ Department of Chemistry, 16748 University of Pavia, Via T. Taramelli 14, 27100 Pavia, Italy; § ROSI Freigeist Juniorgroup, Institut für Physik und Astronomie, 19001 University of Potsdam, Karl-Liebknecht-Str. 24/25, 14476 Potsdam-Golm, Germany

## Abstract

Mobile ions play a key role in the degradation of perovskite solar cells, making their quantification essential for enhancing device stability. Various electrical measurements have been applied to characterize mobile ions. However, discerning between different ionic migration processes can be difficult. Furthermore, multiple measurements at different temperatures are usually required to probe different ions and their activation energies. Here, we demonstrate a new characterization technique based on measuring the thermally activated ion current (TAIC) of perovskite solar cells. The method reveals density, diffusion coefficient, and activation energy of mobile ions within a single temperature sweep and offers an intuitive way to distinguish mobile ion species. We apply the TAIC technique to quantify mobile ions of MAPbI_3_ and triple-cation perovskite solar cells. We find a higher activation energy and a lower diffusion coefficient in the triple-cation devices. TAIC measurements are a simple yet powerful tool to better understand ion migration in perovskite solar cells.

## Graphical abstract

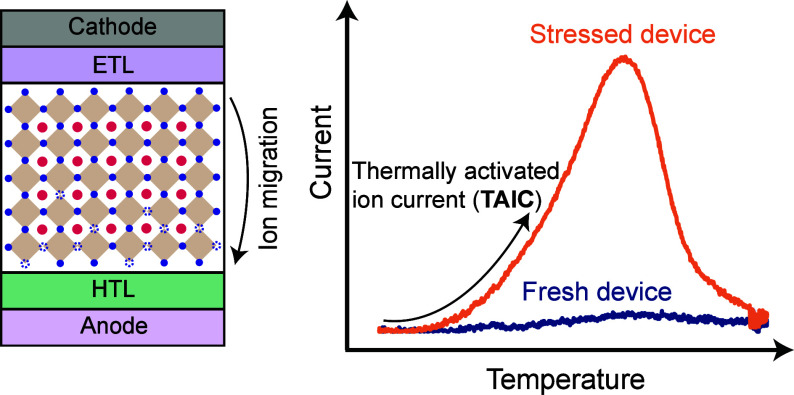

In recent years, mobile ions have been assigned to various losses in perovskite solar cells. They have been attributed to losses in short-circuit current density (*J*_sc_), open-circuit voltage (*V*_oc_), and fill-factor (FF).^1–3^ To understand the impact of mobile ions on device characteristics, quantifying key parameters like the ion density, diffusion coefficient, and activation energy is essential. With the aim of extracting these parameters, various electrical measurements have been applied. The density of mobile ions has been estimated with current transient measurements (also known as bias-assisted charge extraction)^1^ and low-frequency Mott–Schottky measurements.^4,5^ Techniques that are commonly employed to characterize electronic traps in semiconductor materials were transferred to quantify mobile ionic defects in perovskite solar cells, even though their interpretation must be adapted.^6^ These techniques include thermal admittance spectroscopy (TAS)^7^ and deep level transient spectroscopy (DLTS),^8^ which have been employed in efforts to quantify the density, diffusion coefficient, and activation energy of mobile ions.^6,9–12^ All of these techniques can be used to quantify the diffusion coefficient and density of ions within some boundary conditions.^13^ However, multiple measurements at different temperatures are necessary to extract the activation energy of the diffusion coefficient. Additionally, overlapping time constants can make it difficult to discern between different ionic species, especially in transient measurements.

Here, we propose an intuitive technique to quantify the density, diffusion coefficient, and activation energy of mobile ions within a single measurement. The method is inspired by thermally stimulated current (TSC) measurements, which have previously been applied to characterize traps in perovskite solar cells.^14–20^ Similar to current transient measurements, we apply a bias during which mobile ions migrate away from the perovskite/charge transport layer (CTL) interfaces. While applying the bias, we decrease the temperature to 175 K, lowering the diffusion coefficient of the mobile ions. At 175 K, we then remove the applied bias, resulting in an electric field in the perovskite bulk due to the built-in potential of the device. However, because of the low diffusion coefficient of the mobile ions, they do not immediately drift to the interfaces. Mobile ions only begin to drift back to the perovskite/CTL interface when the temperature is increased at a constant rate, resulting in a thermally activated current. To emphasize that we are probing mobile ionic defects, we refer to this as the thermally activated ion current (TAIC).

We use the TAIC technique to quantify mobile ions of two different perovskite solar cells, one with a MAPbI_3_ perovskite and one with a triple-cation perovskite. Figure 1(a) and (b) show the device stack and JV measurements of the MAPbI_3_ device. In Figure 1(c) and (d), the device stack and JV measurements of the triple-cation device are shown. Importantly, we use optimized device stacks for both the triple-cation^21^ and the MAPbI_3_ ^22^ device. This ensures a high shunt resistance, which is necessary to reduce the noise in low-current measurements. Furthermore, we utilize planar CTLs in both devices to avoid complex interface morphologies and to enable modeling of the devices using one-dimensional drift-diffusion simulations. In both devices, we use the self-assembling monolayer MeO-2PACz as the hole transport layer (HTL). For the electron transport layer (ETL), we use PCBM in the case of the MAPbI_3_ perovskite solar cell and C_60_ in the case of the triple-cation device. The surface of the triple-cation perovskite is passivated with a dual passivation of EDAI and PEAI. Details of the fabrication process are available in the Experimental Section.

**Figure 1:**
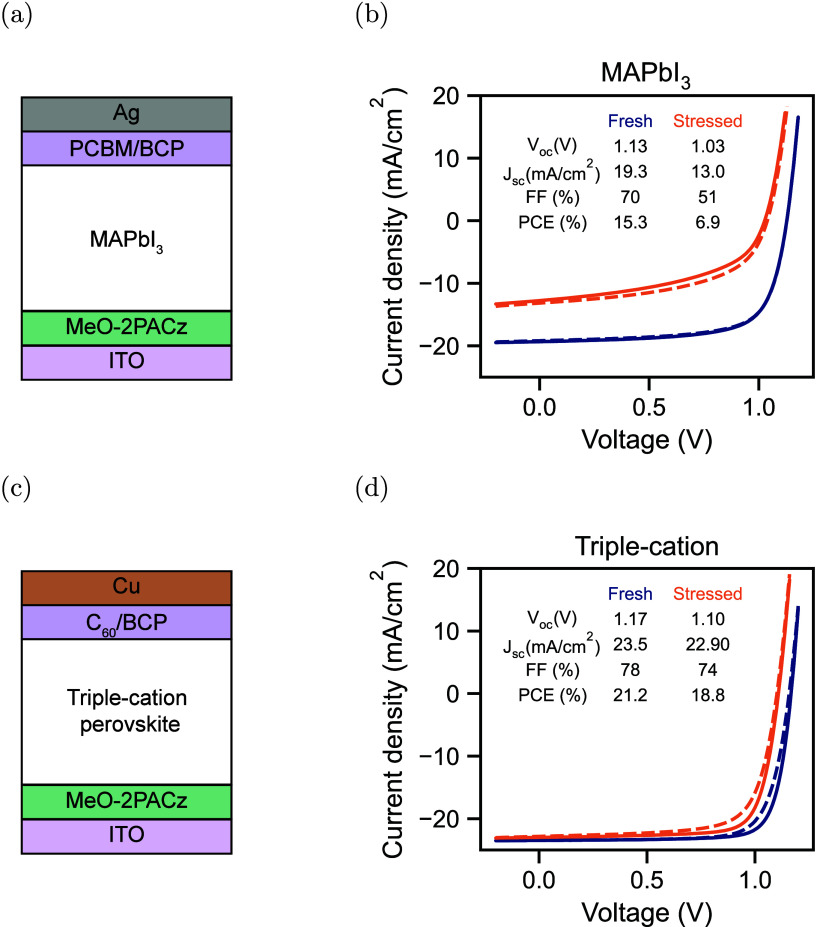
(a) Device stack of the MAPbI_3_ perovskite solar cell. (b) JV measurement of a fresh MAPbI_3_ device and the same device stressed for 32 h at *V*_oc_. (c) Device stack of the triple-cation perovskite solar cell. (d) *JV* measurement of a fresh triple-cation device and the same device stressed for 78 h at *V*_oc_. The dashed lines are the forward, and the solid lines are the reverse voltage scans. The extracted photovoltaic parameters are the mean values of the forward and reverse measurements.

It has previously been demonstrated that stressing perovskite solar cells at *V*_oc_ can lead to degradation by increased ion densities.^1^ We therefore stress the devices at *V*_oc_ under a high-intensity white-light LED with 1-sun equivalent carrier excitation. During stressing, we repeatedly carry out electrical measurements, including JV, capacitance frequency, current transient, and TAIC measurements. For the MAPbI_3_ device, for example, we perform measurements of the fresh device and after 12, 22, and 32 h of stressing at *V*_oc_. The JV measurements are measured under illumination, while the capacitance frequency, current transient, and TAIC measurements are carried out in the dark. Figure 1(b) shows the JV measurement of a fresh MAPbI_3_ device and the same device stressed for a total of 32 h at *V*_oc_, resulting in a significant decrease in *V*_oc_, *J*_sc_, and FF. The triple-cation device is more stable, with a decrease in mainly *V*_oc_ and FF after the maximum stressing time of 78 h at *V*_oc_, as shown in Figure 1(d). The observed degradation in both devices can have numerous origins, including a higher trap density in the bulk or interface^23,24^ or more recombination due to mobile ions.^1^

To quantify the changes related to mobile ions, we carry out TAIC measurements of the devices under the different stressing conditions. The principle of the TAIC measurements is illustrated in Figure 2. At steady state and 0 V applied bias, mobile ions are accumulated at the perovskite/CTL interfaces due to the built-in field of the perovskite. In the first step, we apply a forward bias voltage to the device at 300 K. During this applied bias, mobile ions migrate away from the perovskite/CTL interface into the perovskite bulk, as illustrated in panel A in Figure 2. While still applying the voltage bias, we then decrease the temperature, leading to a decrease in the diffusion coefficient of the mobile ions. Consequently, the ions are ’frozen’ when the voltage pulse is removed and 0 V are applied at 175 K, and they do not drift back to the perovskite/CTL interface, as shown in panel B. In the last step, we slowly increase the temperature with the device at short-circuit. As the temperature increases, the temperature-activated diffusion coefficient of the mobile ions increases exponentially. Consequently, mobile ions start drifting back to the perovskite/CTL interfaces. This results in the thermally activated ion current, which is illustrated in panel C in Figure 2.

**Figure 2:**
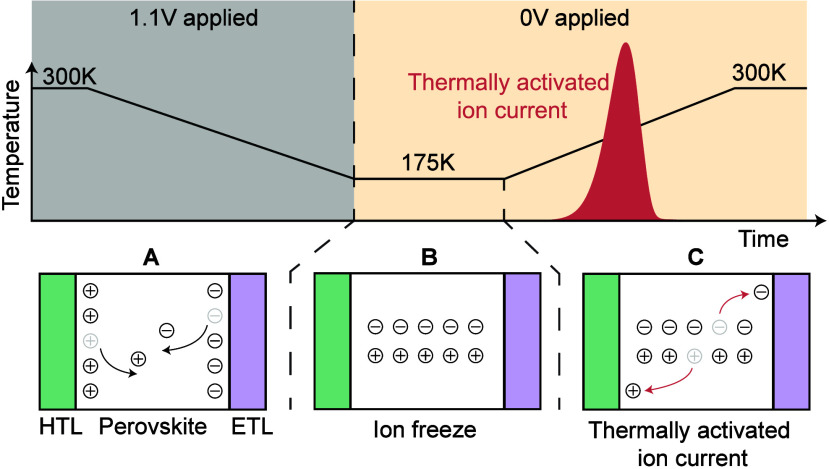
Illustration of the thermally activated ion current measurement. (A) At 300 K, a voltage is applied to the device, during which mobile ions migrate into the perovskite bulk. While the voltage is applied, the temperature is decreased, resulting in a decrease in the ionic diffusion coefficient. (B) At 175 K, the applied voltage is removed, and 0 V are applied. Because of the low temperature, the ions do not drift back to the interface. (C) When the temperature is gradually increased, the diffusion coefficient of the mobile ions increases, resulting in mobile ions drifting back to the perovskite/CTL interfaces, generating the thermally activated ion current (TAIC).

The TAIC measurements of the MAPbI_3_ device and the triple-cation device after different stressing durations are shown in Figure 3(a) and (b), respectively. In both cases, we increase the temperature from 175 to 300 K at a rate of 0.1 K/s and then stabilize at 300 K. Exemplary temperature sweeps are shown as gray lines in Figure 3(a) and (b). We expect the MAPbI_3_ perovskite to be in the tetragonal phase during the entire temperature sweep^25,26^ and see no obvious signs of a phase transition in the triple-cation devices. For both devices, we observe an increase in the current as the temperature increases. At some point, the current peaks and decreases again. For the MAPbI_3_ device, this increase and decrease of the current occur during the temperature sweep. In contrast, the current peak in the triple-cation device occurs only when the temperature sweep is stopped at 300 K. Notably, in both cases, the integral of the current increases with increasing stressing time, suggesting that stressing the devices at *V*_oc_ increases the ion density in both devices. We also measured a second MAPbI_3_ and triple-cation device, yielding similar trends but slightly different absolute values as shown in Figure S3.

**Figure 3:**
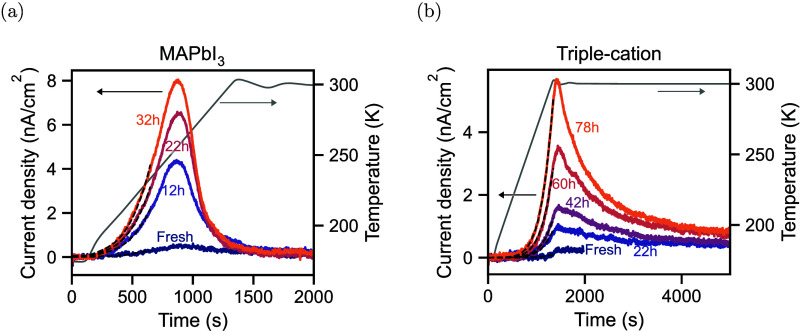
Thermally activated ion current measurements of (a) a MAPbI_3_ and (b) a triple-cation perovskite solar cell for different stressing durations. The black dashed lines represent fits. The gray line represents an exemplary temperature sweep. The extracted ion parameters are shown in Table 1.

We note that for both devices, some charges are extracted immediately after switching the voltage to 0 V at 175 K, as shown in Figure S4­(a) and (b). These could be caused by electrical or fast ionic carriers that are still mobile at low temperatures.

The current *J*_tot_ during the TAIC measurements depends on the density of mobile ions in the bulk *N*_ion,bulk_, the temperature activated diffusion coefficient with prefactor *D*_0,ion_ and activation energy *E*_a_,^27^ and the electric field in the perovskite bulk *E*_bulk_ as

1 $$J_{\mathrm{tot}}\left(t\right)=be^{2}N_{\mathrm{ion},\mathrm{bulk}}\left(t\right)D_{0,\mathrm{ion}}e^{-E_{a}/k_{B}T\left(t\right)}\frac{1}{k_{B}T\left(t\right)}E_{\mathrm{bulk}}\left(t\right)$$

where *b* is a correction factor accounting for the displacement current in the perovskite, *e* is the elementary charge, *k*_B_ is the Boltzmann constant, and *T* is the temperature. A detailed derivation of eq 1 is given in Note 1 in the Supporting Information.

At low temperatures, we can assume that the density of ions in the bulk is constant *N*_ion,bulk_(*t*) = *N*_ion_, because the bulk is not yet depleted of mobile ions. Furthermore, we can approximate the bulk electric field based on an estimated built-in potential of the devices and a potential drop in the transport layers as described in Note 2 in the Supporting Information. With these simplifications, the only unknowns in eq 1 are the activation energy *E*_*a*_ and the product of ion density and diffusion coefficient *N*_ion_ *D*_0,ion_. To extract these variables from the TAIC measurements, we can simply fit eq 1 to the low-temperature part of the TAIC measurements.

To verify this approach, we fit eq 1 to drift-diffusion simulations of TAIC measurements, shown in Figure 4. We note that the drift-diffusion solver we use only allows for a temperature-activated mobility instead of the diffusion coefficient. We therefore extract the temperature-independent prefactor of the ionic conductivity σ_0,ion_ = *eN*_ion_μ_0,ion_, which we can estimate with good accuracy as shown in Table S3.

**Figure 4:**
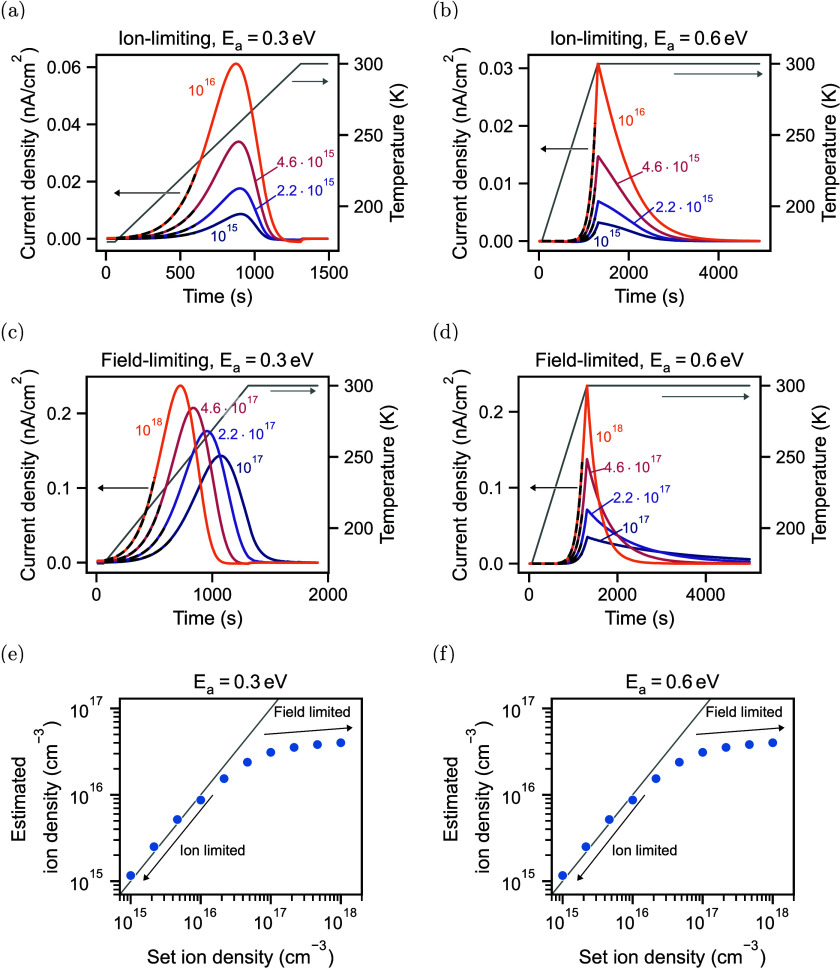
Drift-diffusion simulations of TAIC measurements with different ion densities for the ion-limiting case with (a) an activation energy of 0.3 eV and (b) 0.6 eV and the field limiting case with (c) an activation energy of 0.3 eV and (d) 0.6 eV. All other parameters used for the drift-diffusion simulations are listed in Table S1. The dashed black lines represent fits. (e) Estimated ion density for the simulations with activation energy of 0.3 eV and (f) 0.6 eV. The parameter set used for the simulations is given in Table S1.

For the devices in Figure 3(a) and (b), we assume a built-in voltage of 1 V. With the correction factor described in Note 2 in the Supporting Information, we estimate an electric field of 16 kV/cm for the MAPbI_3_ device and 12 kV/cm for the triple-cation device. The fits of eq 1 are illustrated as dashed lines in Figure 3(a) and (b). We assume that mainly a change in the ion density is responsible for the changes in the TAIC measurements and that the activation energy stays constant across stressing variations. Therefore, we fit the activation energy as a global parameter and the product of density and diffusion coefficient *N*_ion_ *D*_0,ion_ as local parameters. For the MAPbI_3_ and the triple-cation device, we extract activation energies of 0.28 and 0.35 eV, respectively. For comparison, we also fit the activation energy using the natural logarithm of the current, as shown in Figure S5, focusing on both individual trace fits and global fits. The extracted activation energies yield similar results but are slightly lower, which we attribute to the focus of the fit on the higher values and, consequently, traces with more stress when using the exponential fit. Additionally, noise in the measurements with low stress impacts these logarithmic fits more. Based on the fits using eq 2, the extracted values for *N*_ion_ *D*_0,ion_ are listed in Table S4 in the Supporting Information. With the activation energy, *N*_ion_ *D*_0,ion_, and eqs S2–S4 we can now determine the ionic conductivity at different temperatures, for example 300 K, which are listed in Table 1. For both devices, we extract an increasing ionic conductivity with increasing stressing duration, most likely caused by an increasing ion density in the stressed devices. Furthermore, due to the higher activation energy, the ionic conductivity at 300 K of the triple-cation device is 1–2 orders of magnitude lower compared to the MAPbI_3_ device.

**Table 1:** Estimated Values of the Activation Energy *E*_a_, Ionic Conductivity at 300 K σ_ion,300K_, Ion Density *N*_ion_, and Diffusion Coefficient at 300 K *D*_ion,300K_ for the MAPbI_3_ and the Triple-Cation Device at Different Stressing Conditions^a^

device	stressing	*E*_a_ (eV)	σ_ion,300K_ (S/cm)	*N*_ion_ (cm^–3^)	*D*_ion,300K_ (cm^2^/s)
MAPbI_3_	fresh	0.28	3.9 ± 0.3 × 10^–13^	1.8 ± 0.2 × 10^17^	3.5 ± 0.4 × 10^–13^
	12 h		2.6 ± 0.2 × 10^–12^	8.8 ± 0.1 × 10^17^	4.9 ± 0.3 × 10^–13^
	22 h		3.9 ± 0.2 × 10^–12^	10.5 ± 0.1 × 10^17^	6.0 ± 0.3 × 10^–13^
	32 h		5.1 ± 0.3 × 10^–12^	13.7 ± 0.1 × 10^17^	6.0 ± 0.3 × 10^–13^
triple-cation	fresh	0.35	1.0 ± 0.1 × 10^–14^	1.9 ± 1.2 × 10^17^	8.7 ± 5.4 × 10^–15^
	22 h		9.7 ± 0.3 × 10^–14^	15.9 ± 1.2 × 10^17^	9.8 ± 0.7 × 10^–15^
	42 h		14.0 ± 0.5 × 10^–14^	20.9 ± 0.9 × 10^17^	10.8 ± 0.6 × 10^–15^
	60 h		31.8 ± 1.0 × 10^–14^	32.1 ± 0.6 × 10^17^	16.0 ± 0.6 × 10^–15^
	78 h		56.3 ± 1.9 × 10^–14^	43.8 ± 0.5 × 10^17^	20.8 ± 0.7 × 10^–15^

a The values were extracted from the low-temperature fit and the integral of the TAIC measurements. The error of *N*_ion_ is estimated from the minimum detectable ion density based on the noise of the current and the diffusion coefficient at the temperature of the current peaks. The errors of the σ_ion,300K_ correspond to the fitting error. The error of *D*_ion,300K_ is propagated based on the errors of *N*_ion_ and σ_ion,300K_.

To extract the density and diffusion coefficient from *N*_ion_ *D*_0,ion_, we need to determine either the density *N*_ion_ or the diffusion coefficient *D*_0,ion_. In principle, the integral of the TAIC current can be used to determine the ion density. This is, however, only possible as long as the electric field within the perovskite bulk in eq 1 does not significantly change over time. If the electric field is constant, more and more ions drift from the bulk to the perovskite/CTL interfaces, until the bulk becomes depleted of mobile ions, decreasing the current. Then, the integral of the current can be used to approximate the ion density. Because the depletion of ions in the bulk limits the current, we refer to this case as ion-limited.

In contrast, the electric field *E*_bulk_(*t*) in eq 1 can also limit the current. This occurs when ions that accumulate at the interface between perovskite and CTLs screen the built-in potential,^1,28^ decreasing the electric field in the perovskite bulk and therefore the current. In this case, only a fraction of ions drift from the bulk to the interfaces, and the ion density is underestimated when integrating the current.^5^ We refer to this case as the field-limited case.

To illustrate the ion-limited and the field-limited cases, we carried out drift-diffusion simulations of the TAIC measurements for different ion densities and activation energies, which are shown in Figure 4. In the ion limiting case for an activation energy of 0.3 eV, the peak of the TAIC measurements does not shift significantly when the ion density increases, as illustrated in Figure 4(a). For a higher activation energy of 0.6 eV in Figure 4(b), the TAIC currents decay similarly fast for the different ion densities. In contrast to these observations stands the field limiting case. Here, the peak shifts to shorter times for an activation energy of 0.3 eV due to the earlier screening of the built-in field, as shown in Figure 4(c). For an activation energy of 0.6 eV, the earlier screening of the built-in field results in faster decays for higher ion densities, as shown in Figure 4(d). Based on these observations, TAIC measurements can be used to determine if the device suffers from ionic field screening when devices with different ion densities are measured (e.g., during aging).

In the ion-limited case, most mobile ions drift from the bulk to the perovskite/CTL interfaces. We can therefore estimate the ion density by integrating the overall current according to

2 $$N_{\mathrm{ion}}=\frac{1}{b\frac{d_{\mathrm{Pero}}}{2}e}\int_{0}^{\infty }J_{\mathrm{tot}}\left(t\right)dt$$

where *b* is a correction factor accounting for the drop of the potential in the CTLs. Note 2 in the Supporting Information contains details about the correction factor. *d*_Pero_ is the perovskite thickness, and *J*_tot_ is the measured current. The factor $\frac{1}{2}$ originates from the assumption that the mobile ions are distributed homogeneously across the bulk. Then, the average distance that mobile ions migrate is $\frac{d_{\mathrm{Pero}}}{2}$ . When applying eq 2 to drift-diffusion simulations, we can accurately determine the ion density in the ion-limited case, as shown in Figure 4(e) and (f) and Table S3 in the Supporting Information. For increasing ion densities in the field-limited case, the estimated ion density in Figure 4(e) and (f) plateaus, and the extracted ion densities are significantly underestimated. This is consistent with the observation that no electrical measurement can accurately extract ion densities in the field-limited case.^13^ With the ionic conductivity and the density, we can now also determine the mobility of ions in the ion-limited case with good accuracy, as shown in Table S3.

In the measurements in Figure 3(a) and (b), we do not observe a significant shift in the current peak or a faster decay for the more stressed devices. We can therefore assume that the TAIC current is ion-limited. Consequently, we estimate the ion density by integrating the current according to eq 2. We note that the current in the triple-cation device does not fully decay within 5000 s. We therefore extrapolate the data with exponential decays. The estimated ion densities for the different stressing conditions are listed in Table 1. We determine that ion densities for both devices increase by around 1 order of magnitude due to stressing. For the MAPbI_3_ device, the ion density increases from 1.8 × 10^17^ cm^–3^ to 1.4 × 10^18^ cm^–3^. Similarly, stressing the triple-cation device increases the ion density from 1.9 × 10^17^ cm^–3^ to 4.4 × 10^18^ cm^–3^. We can now also determine the diffusion coefficients at 300 K, which are listed in Table 1. For both devices, the diffusion coefficient increases slightly by a factor of 2 due to stressing. Notably, the diffusion coefficient and ionic conductivity of the MAPbI_3_ device are higher than those of the triple-cation device, suggesting that ion migration in the triple-cation devices is suppressed.

We note that, just accounting for the electrostatic effects of mobile ions, we would expect ionic field screening to limit the extracted current for ion densities of 10^17^ cm^–3^ and higher (similar to the drift-diffusion simulations in Figure 4). However, we extract much higher ion density from the TAIC measurements. Possibly, more ions can accumulate at the interface between perovskite and CTLs before the field is screened, which has previously been suggested.^29^ It is also possible that ions recombine when drifting back to the interfaces, not impacting the potential anymore,^30^ or that lateral ion migration^31^ impacts the current, leading to an overestimation of the ion density. Pinpointing the exact cause for the high extracted ion densities is a crucial next step to better understand ion migration, but it is out of the scope of this work.

Interestingly, the extracted diffusion coefficients of both devices are significantly lower than those associated with halide vacancy-mediated ion migration, which is often assumed to be the dominating ionic species. For MAPbI_3_, diffusion coefficients of around 4 ·10^–11^ cm^2^/s up to 10^–9^ cm^2^/s have been previously assigned to iodide vacancy migration.^32–34^ For double cation perovskite solar cells, diffusion coefficients were determined to be in the range of 10^–10^ cm^2^/s.^4^ Possibly, we probe the migration of a slower ionic migration process in the TAIC measurements. To verify this, we carried out capacitance frequency measurements, which allow us to probe faster ionic processes. The resulting capacitance frequency measurements for different stressing times are shown in Figure S6. We observe a rise of the capacitance at around 100 Hz for both the MAPbI_3_ and the triple-cation device in Figure S6­(a) and (b), respectively. A similar capacitance rise has been observed in other capacitance frequency measurements and can be associated with ionic defects.^9,29,34^ Interestingly, the capacitance at lower frequencies increases when the devices are stressed. This suggests the presence of another ionic migration process,^29^ which is slower than the ionic process at around 100 Hz. We can qualitatively reproduce the capacitance increase at around 100 Hz due to a fast ion and the increase of low-frequency capacitance due to a slower ion using drift-diffusion simulations in Figure S7. In the measurements, the process at low frequencies can not be completely resolved with capacitance frequency measurements. However, carrying out capacitance frequency measurements at 360 K shifts the fast defect to higher frequencies and also reveals more of the capacitance increase of the slower ionic defect, as shown in Figure S6­(c) and (d) for the MAPbI_3_ and the triple-cation device, respectively. Additionally, we extracted some current after switching the voltage to 0 V at 175 K, shown in the current transient measurements in Figure S4. Possibly, this current is due to the fast defect observed in the capacitance frequency measurements. From these current transient measurements, we estimate the densities of the fast ion migration process for the MAPbI_3_ and the triple-cation device to be around 10^16^ cm^–3^ and 5 × 10^16^ cm^–3^, see Table S6. These low ion densities are difficult to resolve in TAIC measurements. Altogether, we assign the fast process in the capacitance frequency and low temperature current transients to the migration of halide vacancies. In the TAIC measurements, we then measure the slower ionic migration process, which we probe only partially at low frequencies in the capacitance frequency measurements. The slow ionic process could be due to cation vacancies, which have been associated with lower diffusion coefficients.^35,36^ However, the activation energy associated with cation migration is expected to be around 0.8–1.2 eV,^36–38^ significantly larger than the values found here. A more likely explanation is, therefore, that the fast and slow migration processes are due to different migration pathways of the same ion. It has, for example, been previously reported that ion migration along grain boundaries is significantly faster than migration through perovskite grains.^12,39,40^ Consequently, the current in the TAIC measurements and the slow process in the capacitance frequency measurements could originate from halide vacancies migrating through perovskite grains, whereas the fast process is caused by halide vacancy migration along grain boundaries. To gain a more detailed mechanistic understanding of the origin of the mobile ions, techniques such as time-of-flight secondary ion mass spectrometry^41^ and Kelvin probe force microscopy^30,42^ could further support the TAIC measurements, but are not the focus of this work.

To verify that the TAIC signal is due to ionic carriers, we also carried out the TAIC measurement with an applied voltage of 0 V during the cool-down (using the triple-cation device from Figure 3(b) after 78 h of stressing). Then, as shown in Figure S8­(a), we do not observe any current peak, because no ions were moved into the perovskite bulk. This also verifies that a possible temperature-dependent change of the depletion layers in the CTLs is not the origin of the current. To exclude that we are probing trap emission, we measured the current during heat-up after illuminating a stressed device at low temperatures, while keeping the voltage at 0 V during cool-down. The resulting current in Figure S8­(b) does not show a significant current during the device’s heat-up. If the TAIC measurements were dominated by trap emission, the current profile after a light pulse would result in a similar current profile compared to applying a bias. This is illustrated in Figure S8­(c) using drift-diffusion simulations of a device with traps. The traps are filled using a bias or a light pulse at low temperatures. In both cases, the traps are filled and carriers are emitted from them during the heat-up, resulting in almost the same current profile. As we are not measuring any current after illumination in Figure S8­(b), we can conclude that we are not probing traps and can therefore assign the measured TAIC signal to mobile ions in the perovskite. As additional control measurements, we carried out simple transient current measurements at 300 K, which are shown in Figure S9­(a) and (b) for the MAPbI_3_ and triple-cation device, respectively. The extracted ion densities from the current transients, listed in Table S7, are slightly lower compared to the densities extracted from the TAIC measurements, but follow the same trend, showing higher ion densities for longer stressing times. We attribute the difference in ion density between current transient and TAIC measurements to the shorter measurement duration used for the transient current measurements and the duration of the applied bias, which is longer for the TAIC measurements, possibly resulting in the activation of more ions.

Finally, we carried out TAIC measurements starting and finishing at a higher temperature of 360 K with the aim of observing additional ionic processes. Figure S10­(a) and (b) show exemplary measurements of a MAPbI_3_ and the triple-cation device, respectively. We note that the devices degrade while keeping them at 360 K for extended durations, complicating a controlled stressing profile. In the MAPbI_3_ device, we observe an additional peak and a shoulder at around 310 and 340 K, indicating additional ionic processes. For the triple-cation device in Figure S10­(b), we observe a distinct second peak for which we can extract an activation energy of 0.94 eV. Similarly high activation energies have been previously associated and computationally predicted with the migration of cations in perovskites.^36–38,43^

These measurements illustrate that we can distinguish between different defects within a single temperature sweep. In other techniques like transient current, transient capacitance, and capacitance frequency measurements, multiple measurements at different temperatures are necessary to probe different defects. And even then, it can be difficult to distinguish between different defects in transient measurement, as their characteristic time constants can overlap. The main disadvantage of TAIC measurements is, however, that low ion densities cannot easily be resolved (like the fast defect probed in capacitance frequency measurements). However, once the density of ions is high enough, there is no inherent limitation on which perovskite can be probed as long as the field is not screened and the perovskite is not significantly doped. Highly doped perovskites, such as tin-based ones,^44^ should be treated with caution, as their potential distribution can be vastly different from that of the devices studied in this work.

To clarify the position of TAIC measurements within electrical characterization methods, we summarize the capabilities and limitations of commonly used techniques in Table 2. We note that all the methods can only quantify mobile ion densities if mobile ions do not significantly screen the electric field.^13^

**Table 2:** Comparison of Capabilities and Limitations of Common Electrical Measurement Techniques Used to Quantify Mobile Ions in Perovskite Solar Cells

technique	capabilities	limitations
thermally activated ion current (TAIC)	• Measurement of ion density and diffusion coefficient • Single temperature sweep to determine activation energy • Intuitive measurement to distinguish between different ions	• Difficult to measure low ion densities
transient current^1,4,45,46^	• Measurement of ion density and diffusion coefficient • Slow and fast ions can be measured • Sensitive to low ion densities	• Difficult to distinguish between different ions • Multiple measurements at different temperatures are necessary to determine the activation energy
transient capacitance^10,34,35,47^	• Measurement of ion density and diffusion coefficient • Slow and fast ions can be measured • Sensitive to low ion densities	• A more complex model is necessary to evaluate the data • Difficult to distinguish between different ions • Multiple measurements at different temperatures are necessary to determine the activation energy
capacitance frequency^9,29,34^	• Measurement of ion density and diffusion coefficient • Sensitive to low ion densities	• A more complex model is necessary to evaluate the data • Measurement of slow ions takes a long time • Multiple measurements at different temperatures are necessary to determine the activation energy
low-frequency Mott–Schottky^4,5^	• Measurement of ion density • Sensitive to low ion densities	• Multiple ions can not be measured/distinguished • Does not contain information about the diffusion coefficient

In summary, we have introduced a new measurement technique, thermally activated ion current (TAIC), to characterize mobile ions in perovskite solar cells. TAIC is based on measuring the current due to thermally activated ions. With a simple expression for the TAIC current, we extracted the ionic conductivity by fitting the low-temperature tail of the TAIC measurements. Furthermore, the ion density can be determined by integrating the current in the TAIC measurements, if electric field screening does not limit the overall current. Conveniently, the peak shift in the TAIC data indicates if perovskite solar cells suffer from electric field screening. We applied TAIC measurements to quantify the mobile ion density, diffusion coefficient, and activation energy in a MAPbI_3_ and a triple-cation perovskite solar cell at different stressing conditions. For the MAPbI_3_ device we determined an activation energy of 0.28 eV, mobile ion densities of 1.8 × 10^17^ cm^–3^ to 1.4 × 10^18^ cm^–3^ depending on the stressing condition and a diffusion coefficient of around 5 × 10^–13^ cm^2^/s at 300 K. For the triple-cation device we determined an activation energy of 0.35 eV, mobile ion densities of 1.9 × 10^17^ cm^–3^ to 4.4 × 10^18^ cm^–3^, and a diffusion coefficient of around 10^–14^ cm^2^/s at 300 K, lower than that of the MAPbI_3_ device. We attribute the migration process to halide vacancy migration within perovskite grains. We also observed a faster ionic process in capacitance frequency measurements, which we assign to halide vacancy migration along grain boundaries. Lastly, we showed that it is possible to distinguish between different ionic processes by increasing the temperature range of the TAIC measurements and found a third ion migration process with a high activation energy of 0.94 eV in the triple-cation devices, which we assign to cation migration. In total, TAIC measurements are a promising technique because they are easy to perform, their interpretation is straightforward, and they offer an intuitive visualization of ion migration in perovskite solar cells.

## Experimental Section

### Fabrication of the MAPbI_3_ Devices

The MAPbI_3_ devices were prepared following the procedure described in Pallotta et al.^22^

#### Materials

Chlorobenzene (CB, extra dry, 99.8%), dimethyl sulfoxide (DMSO, ≥99.9% extra dry), *N*,*N*-dimethylformamide (DMF, extra dry, 99.8%), and chloroform (CF, extra dry 99.8%) were purchased from Acros Organics. 2-Propanol (IPA, ≥99.8%), lead iodide (PbI_2_, >98.0%), and MeO-2PACz were purchased from TCI. Methylammonium iodide (MAI, >99.99%) was purchased from GreatCell Solar Materials. Phenyl-C61-butyric acid methyl ester (PCBM, >99.99%) was purchased from Lumatec. Bathocuproine (BCP) was purchased from Sigma-Aldrich. All solutions were prepared in an Ar-filled glovebox, while the deposition of each layer of the solar cell was performed in an N_2_-filled glovebox.

#### Device Fabrication

For the fabrication of the MAPbI_3_ devices, indium tin oxide (ITO)-coated glass substrates (purchased from Yingkou Shangneng Photoelectric material Co.,Ltd.) were consecutively cleaned in acetone and IPA by ultrasonicating for 15 min in each solvent. Substrates were dried with N_2_ airflow and O_2_ plasma treated for 10 min. MeO-2PACz was dissolved in ethanol in a concentration of 0.33 mg/mL and 50 μL were spin-coated onto ITO/glass substrates at 3000 rpm for 30 s and annealed at 100 °C for 10 min. The perovskite precursor solution was prepared by dissolving 0.553 g of PbI_2_ and 0.191 g of MAI powders in 1 mL DMF/DMSO 4/1 v/v solvent. Twenty-five μL of the final solution were deposited on the MeO-2PACz coated substrates and spin-coated with a three-step procedure: the first step proceeded at 1000 rpm (500 rpm/s) for 6 s, the second step proceeded at 5000 rpm for 27 s (2500 rpm/s), while the last step was a speed deceleration of 1250 rpm/s to 0 rpm/s. 150 μL of chlorobenzene were dropped onto the spinning substrate for an antisolvent procedure 6 s after the beginning of the second step. Subsequently, substrates were annealed at 100 °C for 15 min. To fabricate the ETL, PCBM was dissolved in chloroform to produce a 15 mg/mL solution. Twenty μL of the solution were spin-coated at 2000 rpm for 20 s onto the perovskite layer. To prevent the diffusion of the metal contact into the perovskite, 50 μL of 1 mg/mL solution (in isopropanol) of bathocuproine was deposited on PCBM. For all the deposition, a vacuum-based chuck was used. Finally, 80 nm of Ag was thermally evaporated on the device with a shadow mask of 0.0825 cm^2^ area. The evaporation speed was adjusted to 0.01 nm/s for the first 5 nm, 0.02 nm/s from 5 to 15 nm, and 0.06 nm/s for the rest of the procedure.

### Fabrication of the Triple-Cation Devices

#### Solution Preparation

The perovskite solution was prepared by adopting the procedure reported by Seid et al.^21^ PbI_2_ (909.00 mg), FAI (276.06 mg), MABr (3.68 mg), CsI (22.47 mg), and MACl (18.11 mg) were mixed in a DMF/DMSO solvent mixture (5/1 v/v) and stirred for 4 h at 60 °C to form a 1.73 M Cs_0.05_(MA_0.05_FA_0.95_)_0.95_Pb­(I_0.95_Br_0.05_)_3_ perovskite solution. The passivation layers were prepared using high-purity materials from Sigma-Aldrich: PEAI (98%) and EDAI_2_ (>98%). 3.5 mg of PEAI was dissolved in 1 mL of isopropanol (IPA) and sonicated for 30 min. The EDAI_2_ solution was prepared by dissolving 2 mg of EDAI_2_ in a 2 mL 1:1 (v/v) mixture of IPA and toluene

#### Device Fabrication

Planar inverted perovskite solar cells were fabricated using the following layer structure: glass/ITO/MeO-2PACz/Cs_0.05_(MA_0.05_FA_0.95_)_0.95_Pb­(I_0.95_Br_0.05_)_3_/C_60_/BCP/Cu. The fabrication started with ITO-coated glass substrates, which were cleaned sequentially in an ultrasonic bath using acetone, Hellmanex (3% in deionized water), deionized water, ethanol, acetone, and isopropanol, with each solvent being used for 15 min. The cleaned substrates were then exposed to ultraviolet ozone for 30 min before being placed in a nitrogen-filled glovebox.

Next, a MeO-2PACz layer was spin-coated from a 1 mmol ml^–1^ ethanol solution at 3000 rpm for 30 s, followed by annealing at 100 °C for 10 min. Once the substrates had cooled to room temperature, a triple-cation perovskite solution was spin-coated at 4000 rpm for 40 s with a 5 s acceleration time. Seven seconds before the end of the spin-coating process, 300 μL of chlorobenzene was added as an antisolvent, and the perovskite film was annealed at 100 °C for 1 h. For the bilayered passivation, the EDAI_2_ solution was spin-coated onto the perovskite at 5000 rpm for 40 s, and annealed at 100 °C for 10 min. Then, the PEAI solution was spin-coated onto the cooled sample at 5000 rpm for 40 s. Afterward, the samples were transferred to an evaporation chamber where 30 nm of C_60_ was deposited at 0.3 Å/s, followed by 8 nm of BCP and 100 nm of copper, which were evaporated at 0.3Å/s and 0.6 Å/s, respectively, under a high vacuum of 10^–7^ mbar.

### Electrical Characterization

All electrical measurements were carried out in a Janis VPF-100 liquid nitrogen cryostat. During the measurements, the pressure inside the cryostat was around 5 × 10^–6^ mbar.

#### *JV* Measurements

JV measurements were carried out with an Agilent B2902A source-measure unit and a SOLIS-3C high-power white-light LED from Thorlabs. The intensity of the LED was set so that the short-circuit current density of the devices matched with a JV measurement at AM-1.5G illumination (carried out with a Pico solar simulator by G2 V inside a N_2_ filled glovebox).

#### TAIC Measurements

TAIC measurements were carried out using an Agilent B2902A source-measure unit. At 300 or 360 K, a voltage of 1.1 V was applied to the devices. After 60 s, and while still applying the bias, the devices were cooled down and stabilized at 175 K. When switching the voltage to 0 V, the current transient was measured. Then, the temperature was increased to 300 or 360 K and stabilized there, while the current was constantly recorded.

#### Transient Current Measurements

Transient current measurements at 300 K were carried out using an Agilent B2902A source-measure unit. At 300 K, a voltage of 1.1 V was applied to the devices. After 60 s, the voltage was switched to 0 V, and the current was recorded.

#### Capacitance Frequency Measurements

Capacitance frequency measurements were carried out with the MFIA by Zurich Instruments with an AC amplitude of 20 mV in a frequency range of 0.1 Hz to 500 kHz.

### Thickness Measurements

The perovskite film thickness was determined by scratching the films with tweezers and measuring the depth of the scratch with a KLA Tencor P-7 Stylus Profiler.

### Drift-Diffusion Simulations

Drift-diffusion simulations were carried out with Setfos by Fluxim, and the parameter set is listed in Table S1.

## Supplementary Material



## Notes

The parameter set for the drift-diffusion simulations, a note about the correction factor accounting for potential drops in charge transport layers, additional measurements, and extracted ionic parameters (PDF)
