# Supplementary material for: Quantification of Mobile Ions in Perovskite Solar Cells with Thermally Activated Ion Current Measurements
Source: ACS Energy Lett. 2025 Dec 12;11(1):409–18. doi: 10.1021/acsenergylett.5c02224 (PMC12797291; doi:10.1021/acsenergylett.5c02224)
Supplement: Supplementary file 1 [file nz5c02224_si_001.pdf]

Supporting information to: Quantification of mobile ions in perovskite solar cells with thermally activated ion current measurements

Moritz C. Schmidt,[†] Agustin O. Alvarez,[†] Riccardo Pallotta,[‡] Biruk A. Seid,[¶] Jeroen J. de Boer,[†] Jarla Thiesbrummel,[†] Felix Lang,[¶] Giulia Grancini,[‡] and Bruno Ehrler[*,†]

†*LMPV - Sustainable Energy Materials Department, AMOLF, Science Park 104, 1098 XG, Amsterdam, The Netherlands*

‡*Department of Chemistry, University of Pavia, Via T. Taramelli 14, 27100 Pavia, Italy*

¶*ROSI Freigeist Juniorgroup, Institut für Physik und Astronomie, University of Potsdam, Am Neuen Palais 10, 14469 Potsdam, Germany*

E-mail: b.ehrler@amolf.nl

Table S1: Parameters used for the drift-diffusion simulations.

**Parameter**	**Value**
Band gap perovskite $E_{\mathrm{g,Pero}}$ (eV)	1.6
Electron affinity perovskite $E_{\mathrm{aff,Pero}}$ (eV)	3.9
Dielectric constant perovskite $\epsilon_{\mathrm{r,Pero}}$	50
Thickness perovskite d_{Pero} (nm)	500
Effective density of states conduction band perovskite $N_{0,\mathrm{CB,Pero}}$ (cm^{-3})	$2.1 \cdot 10^{18}$
Effective density of states valence band perovskite $N_{0,\mathrm{VB,Pero}}$ (cm^{-3})	$2.1 \cdot 10^{18}$
Mobility electrons in perovskite $\mu_{\mathrm{n,Pero}}$ (cm^2/Vs)	1
Mobility holes in perovskite $\mu_{\mathrm{p,Pero}}$ (cm^2/Vs)	1
Mobile positive ion density in perovskite N_{ion} (cm^{-3})	variable
Immobile negative ion density N_{nion} (cm^{-3})	variable
Prefactor of mobility of ions $\mu_{0,\mathrm{ion}}$ (cm^2/Vs)	variable
Activation energy of mobility of ions E_{a} (eV)	variable
Band gap HTL $E_{\mathrm{g,HTL}}$ (eV)	1.9
Electron affinity HTL $E_{\mathrm{aff,HTL}}$ (eV)	3.4
Dielectric constant HTL $\epsilon_{\mathrm{r,HTL}}$	4.0
Thickness HTL d_{HTL} (nm)	3
Effective density of states conduction band HTL $N_{0,\mathrm{CB,HTL}}$ (cm^{-3})	$2.1 \cdot 10^{18}$
Effective density of states valence band HTL $N_{0,\mathrm{VB,HTL}}$ (cm^{-3})	$2.1 \cdot 10^{18}$
Mobility holes in HTL $\mu_{\mathrm{p,HTL}}$ (cm^2/Vs)	10^{-4}
Acceptor doping density in HTL $N_{\mathrm{A,HTL}}$ (cm^{-3})	0
Band gap ETL $E_{\mathrm{g,ETL}}$ (eV)	2.0
Electron affinity ETL $E_{\mathrm{aff,ETL}}$ (eV)	4.0
Dielectric constant ETL $\epsilon_{\mathrm{r,ETL}}$	5.0
Thickness ETL d_{ETL} (nm)	30

Parameter	Value
Effective density of states conduction band ETL $N_{0,\mathrm{CB,ETL}}$ (cm^{-3})	$2.1 \cdot 10^{18}$
Effective density of states valence band ETL $N_{0,\mathrm{VB,ETL}}$ (cm^{-3})	$2.1 \cdot 10^{18}$
Mobility electrons in ETL $\mu_{\mathrm{n,ETL}}$ (cm^2/Vs)	10^{-4}
Donor doping density in ETL $N_{\mathrm{D,ETL}}$ (cm^{-3})	$1 \cdot 10^{17}$
Work function anode $W_{\mathrm{f,anode}}$ (eV)	5.1
Work function cathode $W_{\mathrm{f,cathode}}$ (eV)	4.1
Applied voltage before TAIC simulation V_{app} (V)	1.1

Supplementary Note 1: TAIC current

Generally, the ionic current J_{ion} can be expressed in terms of the ionic conductivity σ_{ion} and the electric field in the perovskite bulk E_{bulk} as:

$$J_{\mathrm{ion}}(t) = \sigma_{\mathrm{ion}}(t)\, E_{\mathrm{bulk}}(t) \tag{S1}$$

The ionic conductivity is dependent on the ion density $N_{\mathrm{ion,bulk}}$ and mobility of the mobile ions μ_{ion}:

$$\sigma_{\mathrm{ion}}(t) = e\, N_{\mathrm{ion,bulk}}(t)\, \mu_{\mathrm{ion}}(t) \tag{S2}$$

where e is the elementary charge. According to the Nernst-Einstein relation, the mobility depends on the ionic diffusion coefficient D_{ion} as:[1]

$$\mu_{\mathrm{ion}}(t) = \frac{D_{\mathrm{ion}}(t)\, e}{k_{\mathrm{B}} T(t)} \tag{S3}$$

where k_B is the Boltzmann constant and $T(t)$ is the temperature at time t. The diffusion coefficient of mobile ions in perovskites is a temperature-activated process, following:[1]

$$D_\mathrm{ion}(t) = D_\mathrm{0,ion} e^{-\frac{E_\mathrm{a}}{k_\mathrm{B}T(t)}} \tag{S4}$$

where $D_\mathrm{0,ion}$ is a temperature independent prefactor, and E_a is the activation energy associated with the diffusion coefficient. With these relationships, we can define the ionic current in Equation S1 in terms of the mobile ion density and diffusion coefficient:

$$J_\mathrm{ion}(t) = e^2 N_\mathrm{ion,bulk}(t) D_\mathrm{0,ion} e^{-\frac{E_\mathrm{a}}{k_\mathrm{B}T(t)}} \frac{1}{k_\mathrm{B}T(t)} E_\mathrm{bulk}(t) \tag{S5}$$

Finally, the extracted current is the sum of the ionic current and the displacement current. If potential drops in the CTLs, the displacement current results in a lower total current J_tot compared to the ionic current J_ion. We can account for the impact of the displacement current with the correction factor b:

$$J_\mathrm{tot}(t) = b\, J_\mathrm{ion}(t) = b\, e^2 N_\mathrm{ion,bulk}(t) D_\mathrm{0,ion} e^{-\frac{E_\mathrm{a}}{k_\mathrm{B}T(t)}} \frac{1}{k_\mathrm{B}T(t)} E_\mathrm{bulk}(t) \tag{S6}$$

Details about the correction factor are discussed in Supplementary Note 2.

Supplementary Note 2: Correction factor

To simplify the explanation of the correction factor, we use drift-diffusion simulations with the parameter set in Table S1 but undoped transport layers in this section.

Reduction of the bulk electric field

As shown in Equation S5, the drift current of mobile ions to the perovskite/CTL interfaces depends on the electric field in the perovskite bulk. This electric field, in turn, depends on

how much of the built-in potential of the perovskite solar cell drops over the perovskite. If organic CTLs are present, significant parts of the built-in potential can drop in the CTLs due to their low dielectric constant. Then, less potential drops over the perovskite, reducing the electric field in the perovskite bulk. This is illustrated in Figure S1(a), (c), and (e), which show drift-diffusion simulations of the potential of a perovskite solar cell immediately after removing the voltage pulse in a TAIC measurement.

In Figure S1(a), a significantly higher fraction of the potential drops over the ETL compared to Figures S1(c) and (e) due the lower dielectric constant in the ETL ($\epsilon_{\mathrm{r,ETL}} = 1$, $\epsilon_{\mathrm{r,ETL}} = 5$, or $\epsilon_{\mathrm{r,ETL}} = 10$, with $\epsilon_{\mathrm{r,Pero}} = 50$). This difference results in a lower electric field inside the perovskite. We can estimate the potential drop in the perovskite by considering the dielectric constants of the individual layers. We first assume that the electric displacement field D throughout the device is constant:

$$D = \epsilon_0 \epsilon_{\mathrm{r,HTL}} E_{\mathrm{HTL}} = \epsilon_0 \epsilon_{\mathrm{r,Pero}} E_{\mathrm{Pero}} = \epsilon_0 \epsilon_{\mathrm{r,ETL}} E_{\mathrm{ETL}} \tag{S7}$$

where ϵ_0 is the vacuum permittivity and $\epsilon_{\mathrm{r,HTL}}$, $\epsilon_{\mathrm{r,Pero}}$, and $\epsilon_{\mathrm{r,ETL}}$ are the relative dielectric constants of the different layers. E_{HTL}, E_{Pero}, and E_{ETL} refer to the electric field in the individual layers. Next, we express the potential drops in the individual layers in terms of the electric field and the dielectric constant in the perovskite:

$$\Delta V_{\mathrm{HTL}} = E_{\mathrm{HTL}} d_{\mathrm{HTL}} = \frac{\epsilon_{\mathrm{r,Pero}}}{\epsilon_{\mathrm{r,HTL}}} d_{\mathrm{HTL}} E_{\mathrm{Pero}} \tag{S8}$$

$$\Delta V_{\mathrm{Pero}} = E_{\mathrm{Pero}} d_{\mathrm{Pero}} \tag{S9}$$

$$\Delta V_{\mathrm{ETL}} = E_{\mathrm{ETL}} d_{\mathrm{ETL}} = \frac{\epsilon_{\mathrm{r,Pero}}}{\epsilon_{\mathrm{r,ETL}}} d_{\mathrm{ETL}} E_{\mathrm{Pero}} \tag{S10}$$

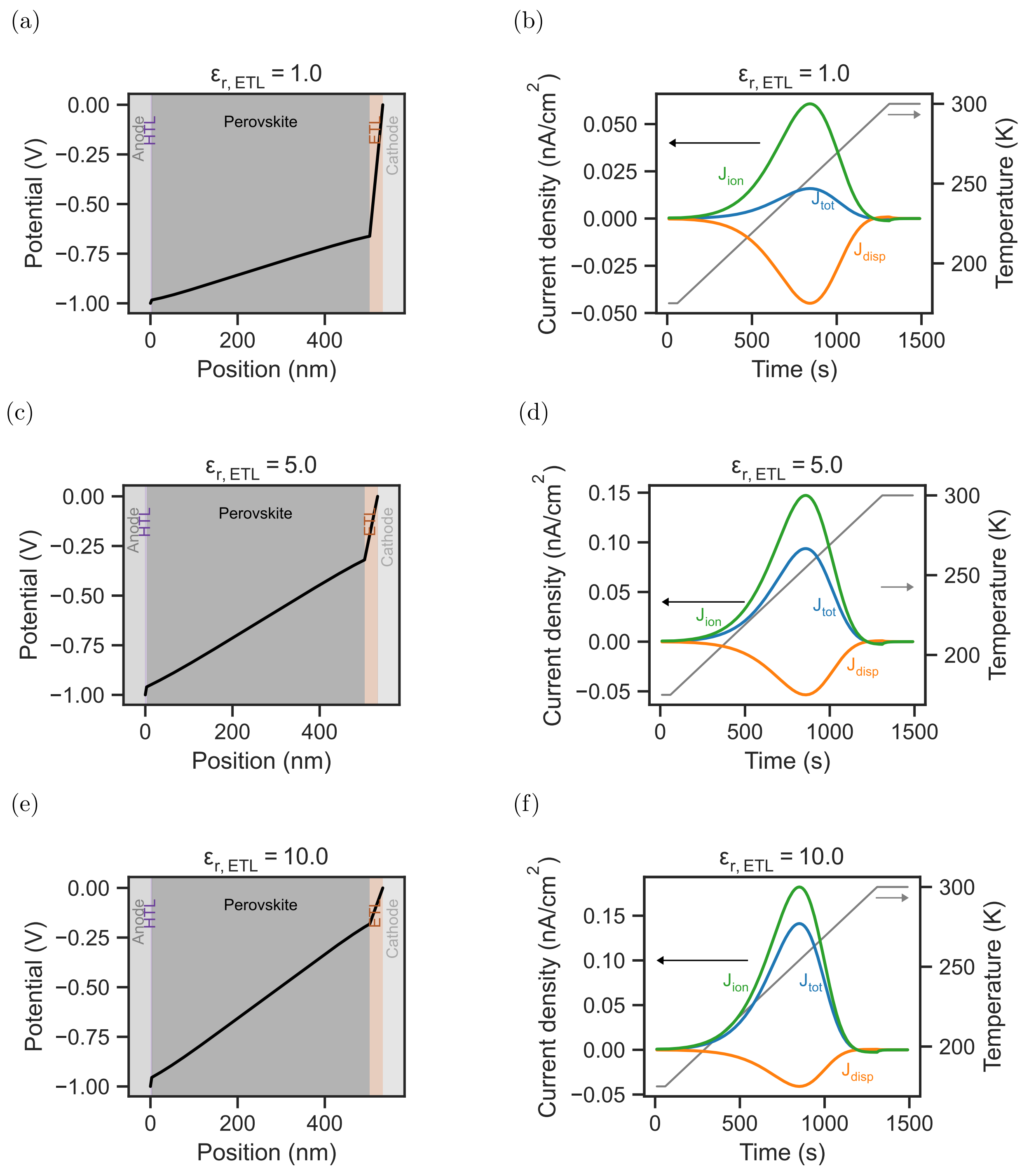

Figure S1: Simulated potential (left column) and current contributions of the ionic current J_{ion} and the displacement current J_{disp} to the total current J_{tot} during the TAIC measurement of a perovskite solar cell (right columns) with different dielectric constants of (a) and (b) $\epsilon_{\mathrm{r,ETL}} = 1.0$, (c) and (d) $\epsilon_{\mathrm{r,ETL}} = 5.0$, and (e) and (f) $\epsilon_{\mathrm{r,ETL}} = 10.0$. The parameter set used for the simulations is given in Table S1. The doping density of the ETL is set to 0.

where d_{HTL}, d_{Pero}, and d_{ETL} are the thicknesses of the different layers. The sum of the potential drops has to equal the built-in voltage V_{bi}:

$$V_{\mathrm{bi}} = \Delta V_{\mathrm{HTL}} + \Delta V_{\mathrm{Pero}} + \Delta V_{\mathrm{ETL}} \tag{S11}$$

$$= E_{\mathrm{Pero}}\big(d_{\mathrm{Pero}} + \frac{\epsilon_{\mathrm{r,Pero}}}{\epsilon_{\mathrm{r,HTL}}} d_{\mathrm{HTL}} + \frac{\epsilon_{\mathrm{r,Pero}}}{\epsilon_{\mathrm{r,ETL}}} d_{\mathrm{ETL}}\big) \tag{S12}$$

We can rearrange this expression for the electric field in the perovskite:

$$E_{\mathrm{Pero}} = V_{\mathrm{bi}}\big(d_{\mathrm{Pero}} + \frac{\epsilon_{\mathrm{r,Pero}}}{\epsilon_{\mathrm{r,HTL}}} d_{\mathrm{HTL}} + \frac{\epsilon_{\mathrm{r,Pero}}}{\epsilon_{\mathrm{r,ETL}}} d_{\mathrm{ETL}}\big)^{-1} \tag{S13}$$

With Equation S9, we can approximate the potential drop in the perovskite:

$$\Delta V_{\mathrm{Pero}} = d_{\mathrm{Pero}} V_{\mathrm{bi}}\big(d_{\mathrm{Pero}} + \frac{\epsilon_{\mathrm{r,Pero}}}{\epsilon_{\mathrm{r,HTL}}} d_{\mathrm{HTL}} + \frac{\epsilon_{\mathrm{r,Pero}}}{\epsilon_{\mathrm{r,ETL}}} d_{\mathrm{ETL}}\big)^{-1} \tag{S14}$$

We define the correction factor b as the fraction of the built-in potential that drops within the perovskite:

$$\begin{aligned} b &= \frac{\Delta V_{\mathrm{Pero}}}{V_{\mathrm{bi}}} \\ &= \big(1 + \frac{\epsilon_{\mathrm{r,Pero}} d_{\mathrm{HTL}}}{\epsilon_{\mathrm{r,HTL}} d_{\mathrm{Pero}}} + \frac{\epsilon_{\mathrm{r,Pero}} d_{\mathrm{ETL}}}{\epsilon_{\mathrm{r,ETL}} d_{\mathrm{Pero}}}\big)^{-1} \end{aligned} \tag{S15}$$

Finally, the bulk electric field can then be calculated using the built-in voltage and the correction factor:

$$E_{\mathrm{Pero}} = \frac{\Delta V_{\mathrm{Pero}}}{d_{\mathrm{Pero}}} = \frac{b V_{\mathrm{bi}}}{d_{\mathrm{Pero}}} \tag{S16}$$

For the cases in Figure S1(a), (c), and (e) and the device parameters in Table S1 (without any doping in the ETL) we calculate a correction factor of 0.25, 0.60 and 0.73, respectively, meaning that the potential drop within the perovskite is approximately 0.25 V, 0.60 V, and

0.73 V (V_{bi} is 1 V). This is in good agreement with the simulations. For the drift-diffusion simulations in the main-text, we use a dielectric constant of $\epsilon_{r,ETL} = 5$ and therefore a correction factor of 0.6 to estimate the bulk electric field based on equation S16

Impact of displacement current

In addition to affecting the electric field in the perovskite bulk, potential drops within the CTLs can also impact the total current measured in TAIC and, more generally, current transient measurements by introducing a displacement current. This is illustrated in Figure S1 for the dielectric constants of $\epsilon_{r,ETL} = 1$, $\epsilon_{r,ETL} = 5$,and $\epsilon_{r,ETL} = 10$ for the ETL. The total extracted current in all cases consists of the ionic current and a displacement current in the perovskite, which are opposite in sign (Note: the ionic current and displacement current plotted here are their overall contributions to the total device current. They are calculated by integrating the position-dependent ionic and displacement currents across the whole device). For a lower dielectric constant of $\epsilon_{r,ETL} = 1$ the displacement current makes up a significant part of the total current J_{tot}, and the total current is only a fraction of the ionic current J_{ion}. This occurs due to the higher potential drop and, consequently, the higher electric field in the ETL. Mobile positive ions that migrate away from the ETL interface result in an increase in the potential drop and, consequently, the electric field in the ETL, which in turn results in a displacement current in the ETL. However, a higher potential drop in the ETL also results in a lower potential drop in the perovskite, which introduces a displacement current opposite the ionic current, which ultimately decreases the total current. To illustrate the relationship between total and ionic current, we have plotted their ratio in Figure S2(a), clearly showing a constant relationship. We also indicated the calculated correction factors for the different dielectric constants as dashed lines, which agree well with the ratios of the currents.

We note, however, that the simple calculation of the correction factor presented here is only valid as long as the potential drops linearly in the device. If, for example, the doping density

of the ETL is large, as shown in Figure S2(a), the ratio between the total and ionic current is no longer constant, and a more complex solution is necessary to correct the current. Other parameters, like the dielectric constant or thickness, also impact the threshold until which this simple correction with the correction factor can be used. In the presented case, we can justify the use of the correction factor because the transport layers are thin and organic, and we therefore expect them to be completely depleted. Then, we can account for the impact of the displacement current on the total current by introducing the correction factor b into Equations 1 and 2 of the main text.

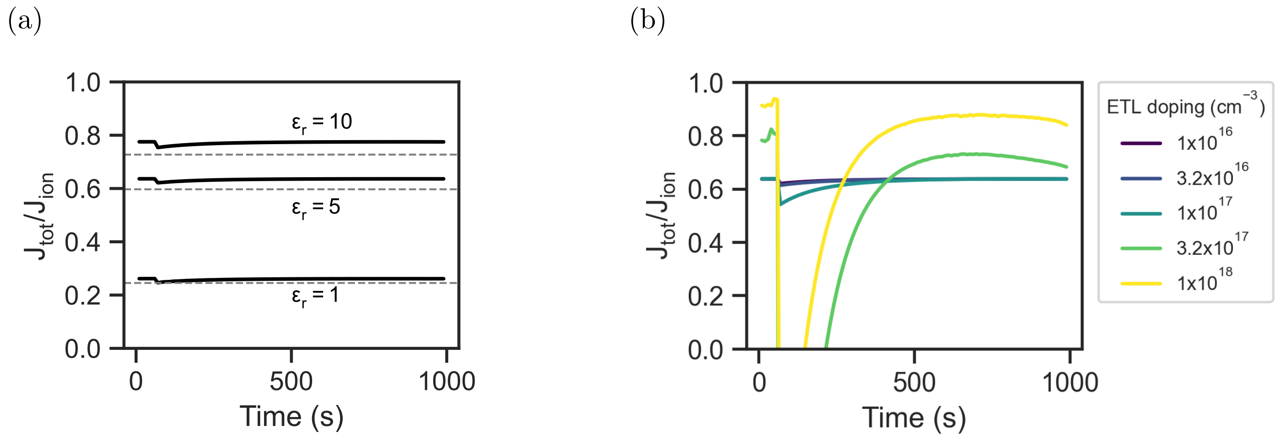

Figure S2: (a) Ratio of the total current J_{tot} and ion current J_{ion} dependent on dielectric constant from Figures S1(b), (d), and (f). The dashed lines represent the estimated ratios based on the correction factor. (b) Ratio of total and ion current of the device with dielectric constant of the ETL of $\epsilon_{\mathrm{r,ETL}} = 5.0$ and different ETL doping densities. Due to the small numerical values of the currents and resulting high noise, we do not plot the ratio for times greater than 1000 s. The parameter set used for the simulations is given in Table S1. In (a) the doping density of the ETL is set to 0.

Calculation of correction factor for measurements

With Equation S15 and the parameters in Table S2, we can estimate the correction factor for the $MAPbI_3$ and the triple-cation device to be 0.76 and 0.66, respectively.

Table S2: Parameter values used to calculate the correction factor of the $MAPbI_3$ and the triple-cation device.

Parameter	$MAPbI_3$	Triple-cation	Comment
Thickness HTL (nm)	2.0	2.0	Estimate
Thickness perovskite (nm)	470.0	550.0	Measured with profilometer
Thickness ETL (nm)	20.0	30.0	Estimate
Dielectric constant HTL	4.0	4.0	Estimate
Dielectric constant perovskite	33.0	43.0	From C-f measurements
Dielectric constant ETL	5.0	5.0	Estimate

Additional information

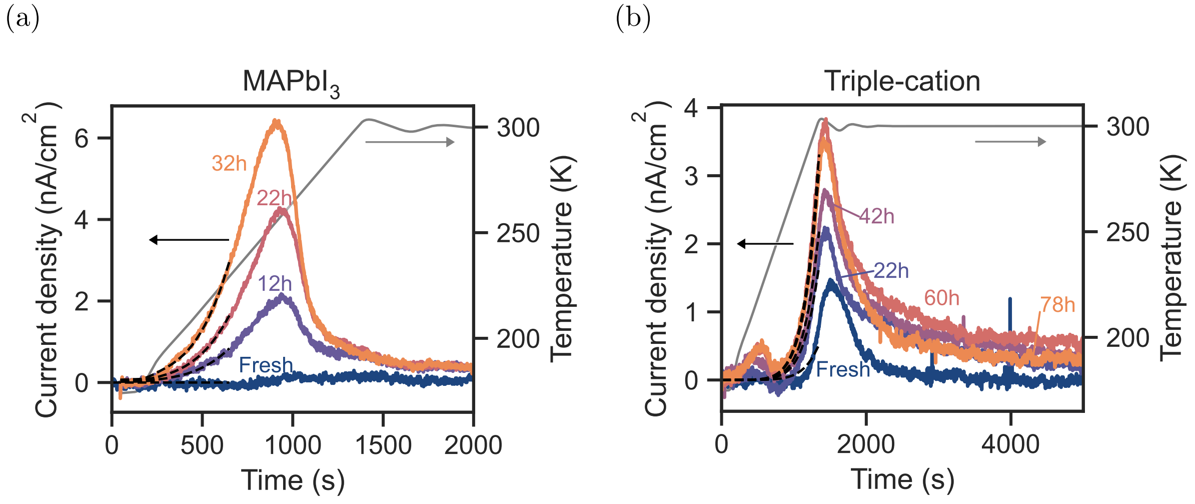

Figure S3: Thermally activated ion current measurements of a second (a) $MAPbI_3$ and (b) triple-cation perovskite solar cell for different stressing durations. The black dashed lines represent fits. The gray line represents an exemplary temperature sweep. The extracted ion parameters are shown in Table S5.

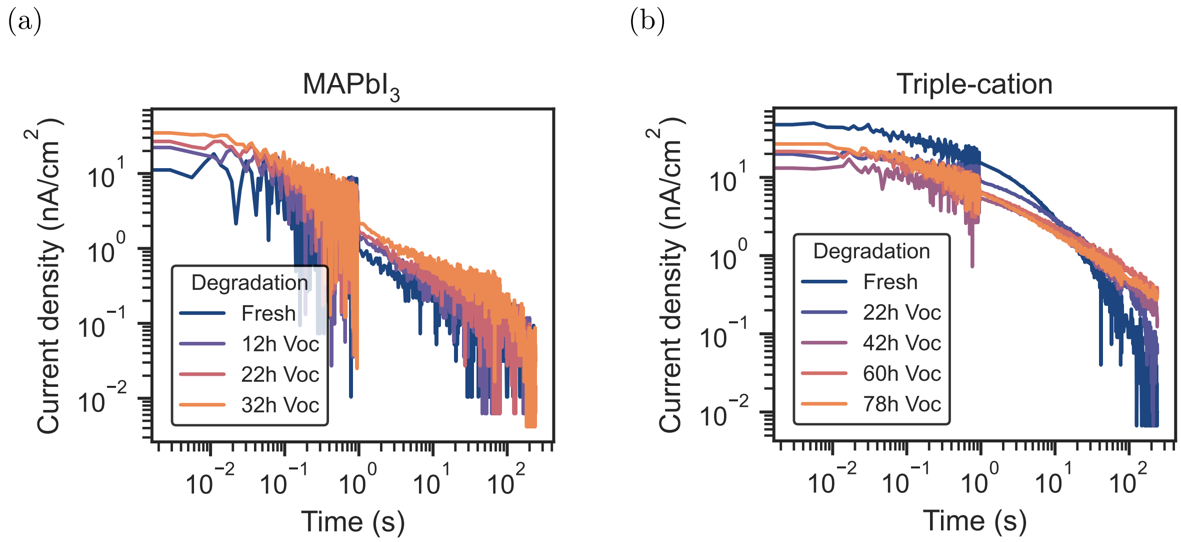

Figure S4: Current transient measurements after switching off the applied bias after cooling the devices down to 175 K in a TAIC measurement of (a) a $MAPbI_3$ device and (b) a triple-cation device. The noise changes around 1 s because the integration time of the source-measure unit changes.

Table S3: Values for the activation energy E_a, ion density N_{ion}, and mobility $\mu_{0,ion}$ extracted from the drift-diffusion simulations in Figure 4. The values were extracted by fitting the low-temperature current and integrating the total current of the TAIC measurements. Because the ion density is significantly underestimated in the field-limited case, we do not determine the ionic mobility.

	Set E_a (eV)	Fit E_a (eV)	Set $\sigma_{0,ion}$ (S/cm)	Fit $\sigma_{0,ion}$ (S/cm)	Set N_{ion} (1/cm^3)	Approx. N_{ion} (1/cm^3)	Set $\mu_{0,ion}$ (cm^2/Vs)	Approx. $\mu_{0,ion}$ (cm^2/Vs)
Ion-limited	0.3	0.29	$1.1 \cdot 10^{-9}$	$7.3 \cdot 10^{-10}$	10^{15}	$1.2 \cdot 10^{15}$	$7 \cdot 10^{-6}$	$3.9 \cdot 10^{-6}$
			$2.4 \cdot 10^{-9}$	$1.6 \cdot 10^{-9}$	$2.2 \cdot 10^{15}$	$2.5 \cdot 10^{15}$		$4.1 \cdot 10^{-6}$
			$5.2 \cdot 10^{-9}$	$3.6 \cdot 10^{-9}$	$4.6 \cdot 10^{15}$	$5.2 \cdot 10^{15}$		$4.4 \cdot 10^{-6}$
			$1.1 \cdot 10^{-8}$	$7.8 \cdot 10^{-9}$	10^{16}	$1.0 \cdot 10^{16}$		$4.8 \cdot 10^{-6}$
	0.6	0.61	$4.8 \cdot 10^{-6}$	$6.5 \cdot 10^{-6}$	10^{15}	$1.2 \cdot 10^{15}$	$3.0 \cdot 10^{-2}$	$3.4 \cdot 10^{-2}$
			$1.0 \cdot 10^{-5}$	$1.6 \cdot 10^{-5}$	$2.2 \cdot 10^{15}$	$2.5 \cdot 10^{15}$		$4.1 \cdot 10^{-2}$
			$2.2 \cdot 10^{-5}$	$3.8 \cdot 10^{-5}$	$4.6 \cdot 10^{15}$	$5.2 \cdot 10^{15}$		$4.5 \cdot 10^{-2}$
			$4.8 \cdot 10^{-5}$	$8.3 \cdot 10^{-5}$	10^{16}	$1.0 \cdot 10^{16}$		$5.1 \cdot 10^{-2}$
Field-limited	0.3	0.28	$1.1 \cdot 10^{-8}$	$4.9 \cdot 10^{-9}$	10^{17}	$2.6 \cdot 10^{16}$	$7.0 \cdot 10^{-7}$	-
			$2.4 \cdot 10^{-8}$	$1.0 \cdot 10^{-8}$	$2.2 \cdot 10^{17}$	$3.4 \cdot 10^{16}$		-
			$5.2 \cdot 10^{-8}$	$2.1 \cdot 10^{-8}$	$4.6 \cdot 10^{17}$	$3.8 \cdot 10^{16}$		-
			$1.1 \cdot 10^{-7}$	$4.4 \cdot 10^{-8}$	10^{18}	$4.0 \cdot 10^{16}$		-
	0.6	0.56	$4.8 \cdot 10^{-5}$	$1.1 \cdot 10^{-5}$	10^{17}	$3.1 \cdot 10^{16}$	$3.0 \cdot 10^{-3}$	-
			$1.0 \cdot 10^{-4}$	$2.5 \cdot 10^{-5}$	$2.2 \cdot 10^{17}$	$3.5 \cdot 10^{16}$		-
			$2.2 \cdot 10^{-4}$	$5.3 \cdot 10^{-5}$	$4.6 \cdot 10^{17}$	$3.8 \cdot 10^{16}$		-
			$4.8 \cdot 10^{-4}$	$1.1 \cdot 10^{-4}$	10^{18}	$4.0 \cdot 10^{16}$		-

Table S4: Fitting value of the product of ion density and diffusion coefficient prefactor $N_{\mathrm{ion}}D_{0,\mathrm{ion}}$ extracted from the low temperature fits in Figure 3. The errors correspond to the fitting error.

Device	Stressing	$N_{\mathrm{ion}}D_{0,\mathrm{ion}}(\mathrm{cm\,s})^{-1}$
$MAPbI_3$	Fresh	$2.7 \pm 0.2 \cdot 10^{9}$
	12h	$1.8 \pm 0.1 \cdot 10^{10}$
	22h	$2.7 \pm 0.2 \cdot 10^{10}$
	32h	$3.5 \pm 0.2 \cdot 10^{10}$
Triple-cation	Fresh	$1.5 \pm 0.1 \cdot 10^{9}$
	22h	$1.4 \pm 0.1 \cdot 10^{10}$
	42h	$2.0 \pm 0.1 \cdot 10^{10}$
	60h	$4.5 \pm 0.1 \cdot 10^{10}$
	78h	$8.0 \pm 0.3 \cdot 10^{10}$

Table S5: Estimated values of the activation energy E_{a}, ionic conductivity at 300 K $\sigma_{\mathrm{ion,300K}}$, ion density N_{ion}, and diffusion coefficient at 300 K $D_{\mathrm{ion,300K}}$ for the second $MAPbI_3$ and the triple-cation devices in Figure S3. The values were extracted from the low-temperature fit and the integral of the TAIC measurements. The error of N_{ion} is estimated from the minimum detectable ion density based on the noise of the current and the diffusion coefficient at the temperature of the current peaks. The errors of the $\sigma_{\mathrm{ion,300K}}$ correspond to the fitting error. The error of $D_{\mathrm{ion,300K}}$ is propagated based on the errors of N_{ion} and $\sigma_{\mathrm{ion,300K}}$. For the fresh $MAPbI_3$ device, we could not extract any values because the current is below the noise.

Device	Stressing	E_{a} (eV)	$\sigma_{\mathrm{ion,300K}}$ (S/cm)	N_{ion} (cm^{-3})	$D_{\mathrm{ion,300K}}$ (cm^2/s)
$MAPbI_3$	Fresh	0.25	-	-	-
	12h		$9.0 \pm 0.7 \cdot 10^{-13}$	$5.8 \pm 0.2 \cdot 10^{17}$	$2.5 \pm 0.2 \cdot 10^{-13}$
	22h		$1.8 \pm 0.1 \cdot 10^{-12}$	$9.3 \pm 0.2 \cdot 10^{17}$	$3.1 \pm 0.2 \cdot 10^{-13}$
	32h		$3.2 \pm 0.2 \cdot 10^{-12}$	$12.1 \pm 0.1 \cdot 10^{17}$	$4.2 \pm 0.3 \cdot 10^{-13}$
Triple-cation	Fresh	0.42	$5.7 \pm 0.8 \cdot 10^{-14}$	$2.9 \pm 0.3 \cdot 10^{17}$	$3.1 \pm 0.5 \cdot 10^{-14}$
	22h		$1.8 \pm 0.2 \cdot 10^{-13}$	$13.1 \pm 0.4 \cdot 10^{17}$	$2.2 \pm 0.3 \cdot 10^{-14}$
	42h		$2.4 \pm 0.3 \cdot 10^{-13}$	$19.1 \pm 0.5 \cdot 10^{17}$	$2.0 \pm 0.3 \cdot 10^{-14}$
	60h		$3.6 \pm 0.5 \cdot 10^{-13}$	$27.1 \pm 0.5 \cdot 10^{17}$	$2.1 \pm 0.3 \cdot 10^{-14}$
	78h		$3.4 \pm 0.4 \cdot 10^{-13}$	$24.2 \pm 0.5 \cdot 10^{17}$	$2.2 \pm 0.3 \cdot 10^{-14}$

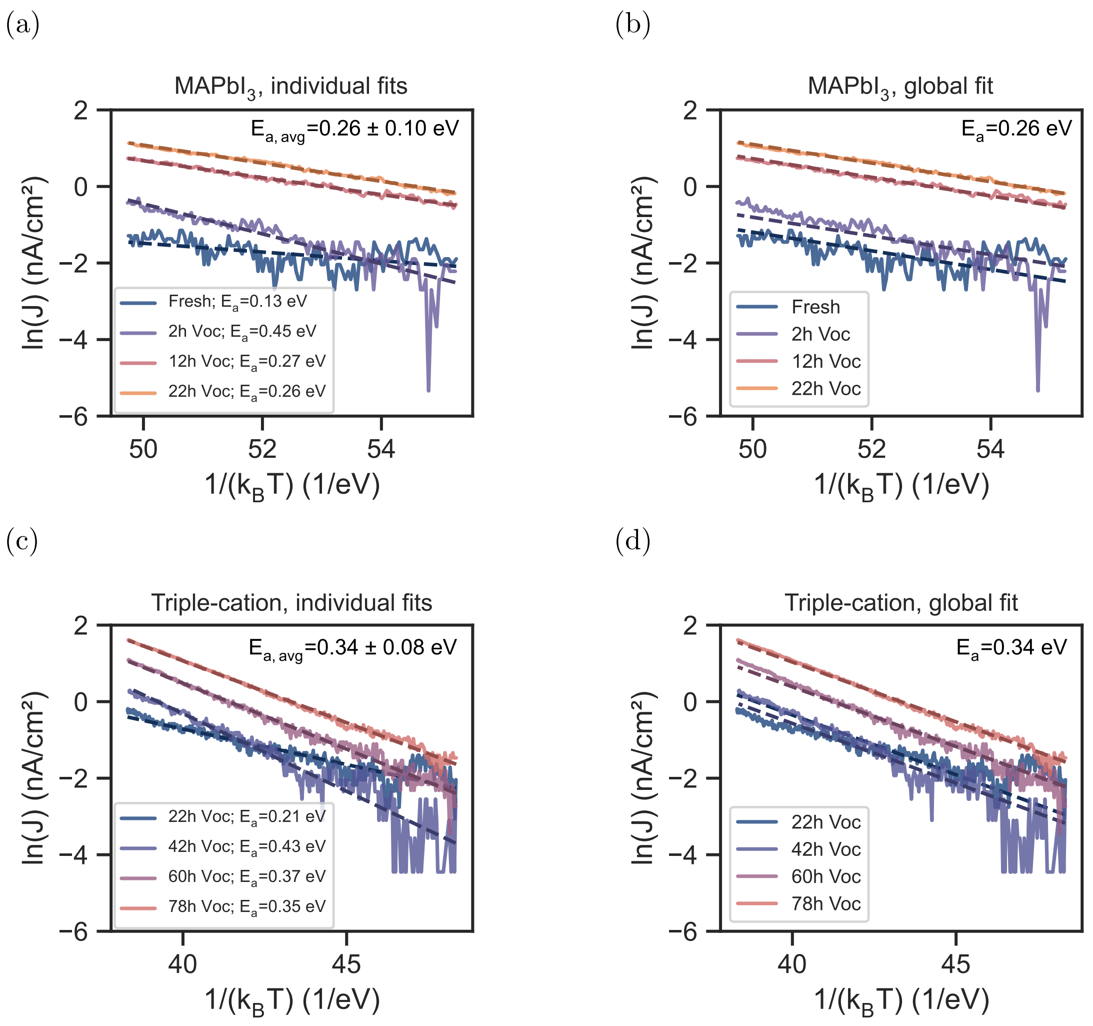

Figure S5: Individual fits (left column) and global fits (right column) of (a) and (b) the $MAPbI_3$ device and (c) and (d) the triple-cation device from Figure 2 of the main text. The activation energy is extracted from fitting the function $\ln(J) = -E_a x + \ln(\frac{bx}{k_B})$ with $x = \frac{1}{k_B T}$ and the Boltzmann's constant k_B and the constant variable b. For the triple-cation device, we ignore the fresh device because the current is too low and noisy. Slight differences in the global fits compared to using equation 1 for the fitting originate from the different weighing. When using equation 1, larger values of the exponentials have a greater impact on the fits.

Table S6: Ion density estimated from current transient measurements measured at 175 K in Figure S4.

Device	Stressing	$N_{\text{ion}}(\text{cm}^{-3})$
$MAPbI_3$	Fresh	$9.8 \cdot 10^{15}$
	12h	$1.2 \cdot 10^{16}$
	22h	$1.3 \cdot 10^{16}$
	32h	$1.8 \cdot 10^{16}$
Triple-cation	Fresh	$4.8 \cdot 10^{16}$
	22h	$5.5 \cdot 10^{16}$
	42h	$5.1 \cdot 10^{16}$
	60h	$6.8 \cdot 10^{16}$
	78h	$5.7 \cdot 10^{16}$

Table S7: Ion density estimated from current transient measurements measured at 300 K in Figure S9.

Device	Stressing	$N_{\text{ion}}(\text{cm}^{-3})$
$MAPbI_3$	Fresh	$5.8 \cdot 10^{16}$
	12h	$5.2 \cdot 10^{17}$
	22h	$7.4 \cdot 10^{17}$
	32h	$9.1 \cdot 10^{17}$
Triple-cation	Fresh	$2.5 \cdot 10^{16}$
	22h	$1.4 \cdot 10^{17}$
	42h	$3.4 \cdot 10^{17}$
	60h	$6.3 \cdot 10^{17}$
	78h	$9.0 \cdot 10^{17}$

(a) (b)

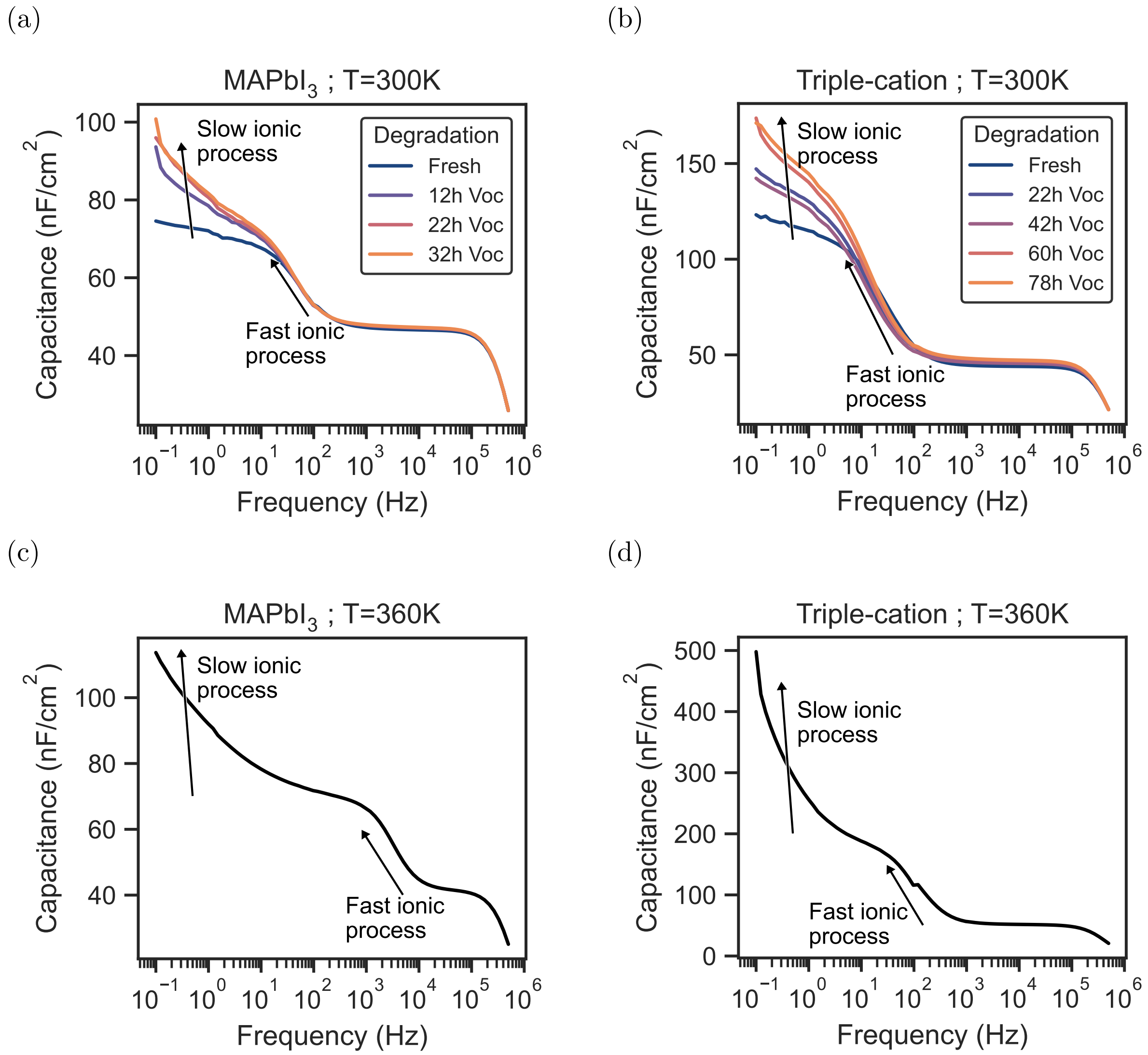

Figure S6: Capacitance frequency measurements of (a) a $MAPbI_3$ device at 300 K after different stressing durations at V_{oc}, (b) a triple-cation device at 300 K after different stressing durations at V_{oc}, (c) a $MAPbI_3$ device at 360 K and (d) a triple-cation device at 360 K.

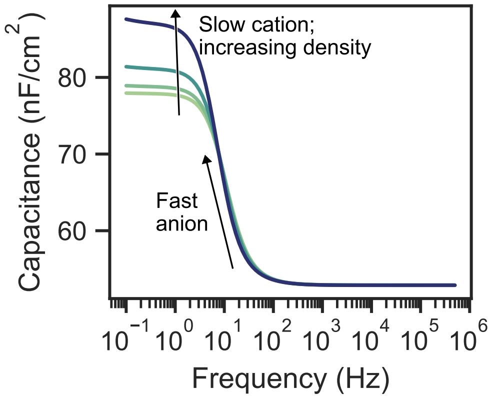

Figure S7: Capacitance frequency measurements using the parameters in Table S1 at 300 K with an additional mobile anion of density $3 \cdot 10^{16}\,\mathrm{cm}^{-3}$ and mobility $8 \cdot 10^{-8}\,\mathrm{cm}^2/\mathrm{Vs}$. The cation density is swept from $10^{15}\,\mathrm{cm}^{-3}$ to $10^{16}\,\mathrm{cm}^{-3}$.

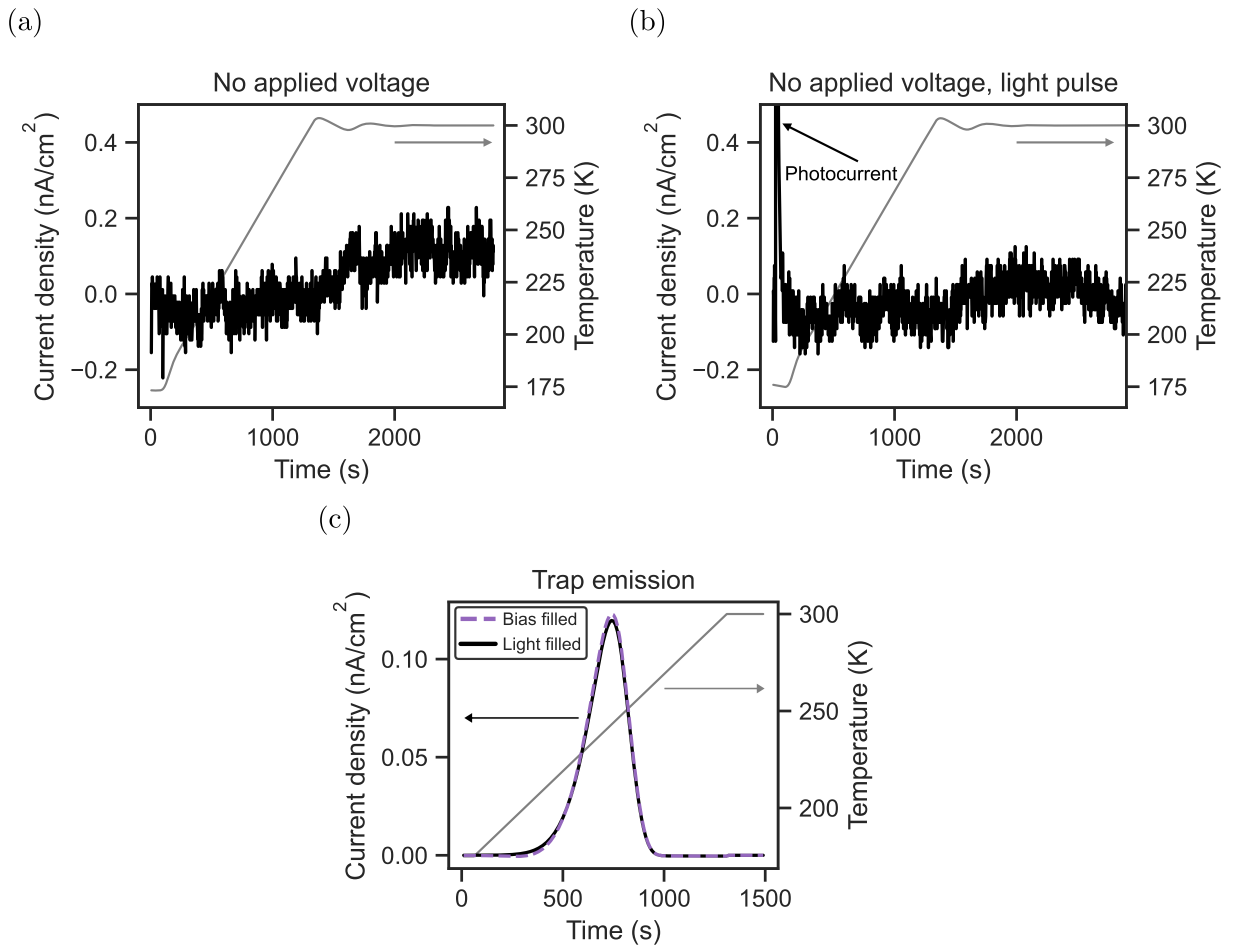

Figure S8: TAIC measurement of stressed triple-cation device (a) without any applied voltage during the cool-down and (b) without any applied voltage during the cool-down and briefly illuminated at 175 K. (c) Drift-diffusion simulation with parameters in Table S1 but with traps instead of ions. The trap density is $10^{16}\,cm^{-3}$, the trap depth 0.5 eV, and the electron and hole capture rates are $10^{-10}\,cm^3/s$. The traps are either filled by applying a voltage bias of 1.1 V (bias filled) or a light pulse at low temperatures (light filled).

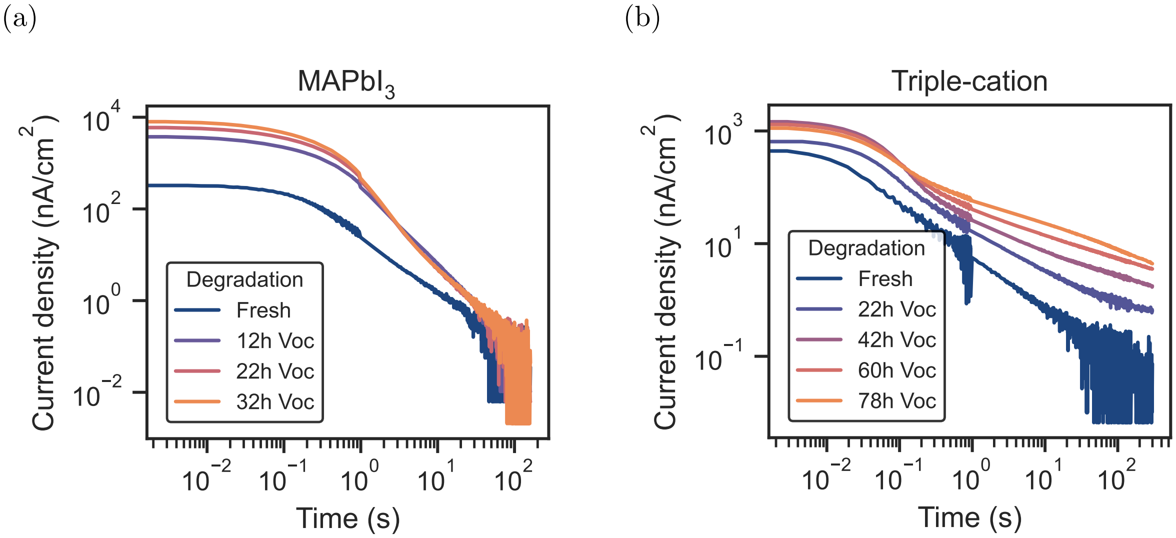

Figure S9: Current transient measurements at 300 K of (a) a $MAPbI_3$ device and (b) a triple-cation device. The noise changes around 1 s because the integration time of the source-measure unit changes. To avoid the devices recovering before starting the TAIC measurements, we limit the measurement time of the transient current measurements.

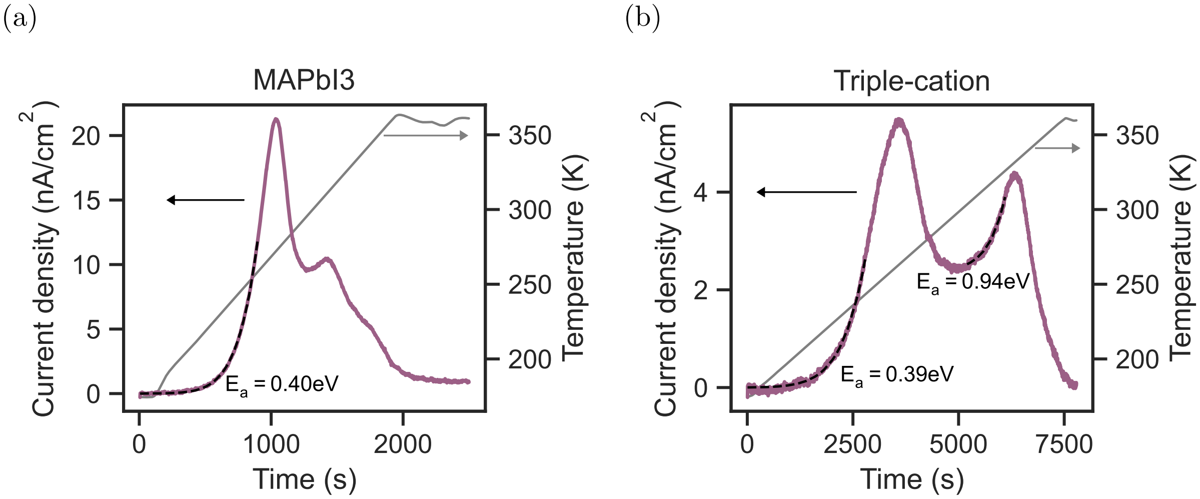

Figure S10: TAIC measurements starting and ending at 360 K of (a) a $MAPbI_3$ and (b) a triple-cation device. The temperature sweep speed for the $MAPbI_3$ device is 0.1 K/s and for the triple-cation device 0.025 K/s. Dashed lines indicate fits.

References

(1) Futscher, M. H.; Milić, J. V. Mixed Conductivity of Hybrid Halide Perovskites: Emerging Opportunities and Challenges. *Frontiers in Energy Research* **2021**, *9*, 629074.
